# Quinine in Gynecic and Obstetric Practice

**Published:** 1882-01

**Authors:** 


### QUININE IN GYNECIC AND OBSTETRIC PRACTICE.

Prof. Henry F. Campbell of Augusta, Ga., presents the following conclusions in a paper read before the Fifth Annual Meeting of the American Gynecological Society upon the use of Quinine in Obstetrics and Gynecology:

*First.* That an exalted reflex excitability of the cerebro-spinal centres, as well as general plethora, may be recognized as a characteristic condition of the pregnant woman from the date of conception to the completion of involution. They may be termed "the gravid development and exaltation of the nerve-centres."

*Second.* That this provisionally increased development and polarity, intended for the purposes of fetal and uterine growth, renders the woman, during its continuance, eminently liable to become the subject of various morbid reflexes more or less peculiar to her condition.

*Third.* That these morbid reflexes are of two perfectly distinct and dissimilar kinds, differing widely as they may happen to occur, before or after parturition.

*Fourth.* During the entire period of pregnancy, and until after laboı, the reflexes are of an *excito-motory* character, restricted to the muscular apparatuses of the uterus and of general volition. They are apyretic and non-inflammatory. Their paroxysms threaten premature expulsion of the fetus in pregnancy, and eclamptic convulsions in labor.

*Fifth.* After parturition, the reflexes are of an *excito-secretory* character. They are propagated, through the ganglionic or vaso-motor nerves, to the blood-vessels and capillaries of the pelvic organs and tissues and of the general system. They are marked by fever, congestion, and inflammation, with their products and consequences. Septic fever and peritonitis, with arrest of involution and mammary abscess, are their not uncommon results.

*Sixth.* That quinine, by its contractile action on the capillaries of the cerebro-spinal centres, exsanguinates their nervous structure and, more than any known agent, depresses the reflex excitability from which the varied morbid phenomena of both pregnancy and child-bed originate.

*Seventh.* That quinine, except in cases of idiosyncrasy or from an injudicious administration of the agent, exercises no influence whatever to superinduce premature expulsion of the fetus.

*Eighth.* That moderate cinchonism adjusted to the type and approach of the paroxysmal neuroses, which endanger the welfare of the fetus during pregnancy, is one of our most efficient resources in many cases of threatened abortion and of premature labor. During parturition it may give "steadiness" to irregular uterine contractions; and, continued during labor, cinchonism is, in a most valuable degree, prophylactic against threatened eclampsia.

*Ninth.* That the reflexes of child-bed pertaining as they do, primarily and principally, to the recently evacuated uterus—well likened to an organ in a traumatic condition—opportune and ready for the awakening of fever and inflammation, are of the gravest character, frequently tending to disorganization and death, or else to permanent and irreparable injury. These "reflexes" constitute a dreaded class of diseases, most commonly called "puerperal," which, by universal consent, must be *prevented* rather than trusted to efforts, so often unavailing, for their cure. To this end, the most valuable and reliable prophylactic method will be found to consist in *the daily administration of quinine to the degree of moderate cinchonism* from the day of parturition and to be continued daily until normal involution is safely secured. By the observance of this "routine" as a rule it is believed that the occurrence of puerperal diseases will be largely prevented, and that the rate of child-bed mortality will be greatly diminished.

*Tenth.* That cinchonism in its quality of preventing and controlling inflammation, whether traumatic or idiopathic, and of suppressing suppuration—all of which is due to its power over reflex excitability of the cord and its action on the capillaries—has a claim to antiseptic value superior to Listerism, and is less to be dispensed with than carbolic acid or any of the means and appliances of the recognized "antiseptic method." In general surgery and especially in uterine surgery, as well as after parturition, the combination of carbolized irrigations and applications to diminish *peripheral* excitability with persistent *cinchonism* to depress *centric* excitability should constitute hereafter an antiseptic method more reliable, generally practicable, and less to be dispensed with than the most faithful observance of the complex Listerian process.

